# Potential of exosomes as diagnostic biomarkers and therapeutic carriers for doxorubicin-induced cardiotoxicity

**DOI:** 10.7150/ijbs.58786

**Published:** 2021-03-27

**Authors:** Chao Tian, Yanyan Yang, Baochen Bai, Shizhong Wang, Meixin Liu, Rui-Cong Sun, Tao Yu, Xian-ming Chu

**Affiliations:** 1 Department of Cardiology, The Affiliated Hospital of Qingdao University, Qingdao 266000, China.; 2 Department of Immunology, Basic Medicine School, Qingdao University, Qingdao 266071, China.; 3 Institute for Translational Medicine, The Affiliated Hospital of Qingdao University, Qingdao 266021, China.; 4 Department of Cardiac Ultrasound, The Affiliated hospital of Qingdao University, Qingdao 266000, China.; 5 Department of Cardiology, The Affiliated Cardiovascular Hospital of Qingdao University, Qingdao 266032, China.; 6 Department of Cardiovascular Surgery, The Affiliated Cardiovascular Hospital of Qingdao University, Qingdao 266000, China.

**Keywords:** doxorubicin, cardiotoxicity, exosome, biomarker, delivery vehicles

## Abstract

Doxorubicin (DOX) is a kind of representative anthracyclines. It has greatly prolonged lifespan of cancer patients. However, a long course of DOX chemotherapy could induce various forms of deaths of cardiomyocytes, such as apoptosis, pyroptosis and ferroptosis, contributing to varieties of cardiac complications called cardiotoxicity. It has become a major concern considering the large number of cancer patients' worldwide and increased survival rates after chemotherapy. Exosomes, a subgroup of extracellular vesicles (EVs), are secreted by nearly all cells and consist of lipid bilayers, nucleic acids and proteins. They can serve as mediators between intercellular communication via the transfer of bioactive molecules from secretory to recipient cells, modulating multiple pathophysiological processes. It has been proven that exosomes in body fluids can serve as biomarkers for doxorubicin-induced cardiotoxicity (DIC). Moreover, exosomes have attracted considerable attention because of their capacity as carriers of certain proteins, genetic materials (miRNA and lncRNA), and chemotherapeutic drugs to decrease the dosage of DOX and alleviate cardiotoxicity. This review briefly describes the characteristics of exosomes and highlights their clinical application potential as diagnostic biomarkers and drug delivery vehicles for DIC, thus providing a strategy for addressing it based on exosomes.

## Introduction

Globally, malignancies are a dominant cause of death and chemotherapy is an effective strategy. There are varieties of chemotherapeutic drugs, mainly including anthracycline, alkylating agent, antimetabolie antineoplastic agents, and antitumor antibiotics. Doxorubicin is a kind of typical anthracyclines, which belongs to the antitumor antibiotics. However, a long course of DOX chemotherapy exerts dose-dependent side effects on cardiomyocytes due to their vulnerability and low regenerative abilities 1, 2. These side effects were called cardiotoxicity.

The emergence of cardiotoxicities, such as arrhythmia, myelosuppression, cardiomyopathy, and congestive heart failure (CHF) limits clinical use of DOX 3, of which CHF is the most severe complication of long-term DOX chemotherapy 4. Elucidating the mechanisms of DIC can unveil possible targets for solving these troubles. However, the exact mechanism remains unclear. The pivotal role of reactive oxygen species (ROS) or oxidative stress in cardiotoxicity has been highlighted 5-7. They are involved in senescence 8 and various deaths of cardiomyocyte induced by DOX, including apoptosis 9, autophagy 10, necroptosis 11, pyroptosis 12, and ferroptosis 13. Non-coding RNAs, which were previously considered as “evolutionary junk”, were demonstrated to crucially participate in cardiovascular diseases (CVD) 14-21, particularly they exert significant influence during DIC 22. Collectively, these findings explain the riddle of DIC to some extent.

Although accumulating studies have explored the means for DIC 23-26, dexrazoxane (DXR) remains the only FDA-approved drug for it 27. And more powerful agents besides DXR need to be explored to deal with this troublesome problem. Moreover, early detection contributes to early intervention and improving prognosis. But it still lacks specific means for detecting DIC. Exosomes, secreted by most cells, lie in various body fluid and have been explored as biomarkers for many diseases 28. Besides, they can transport bioactive molecules to regulate many physiological activities 29, 30 and have the potential as delivery vehicles of drugs and regulatory molecules for CVD 31, tumors 32, and inflammation 33. Notably, exosomes can protect their cargos from degradation due to their double-layered membrane, prolonging its half-life in circulation and enhancing its therapeutic functions.

In this review, we point out the possibility of exosomes as biomarkers and delivery vehicles for diagnosis and therapy for DIC. We hope that with deepening cognition to exosomes' potential in DIC, they can come into clinical practice in the near future.

## Definition, biogenesis, and structure of exosomes

Secreted by membrane vesicles, extracellular vesicles (EVs) are highly heterogeneous and traditionally divided into three categories based on size: apoptosomes, microvesicles, and exosomes. Exosomes, derived from the endosomal membrane through endocytosis, range from 30 to 150 nm 34, 35. They are present in almost all body fluids 36, 37. The formation of exosomes undergoes double invagination. First, the plasma membrane invaginates to form a cup-shaped structure called the early-sorting endosome (ESE) with the assistance of Golgi complex. The endoplasmic reticulum also participates in this process by providing the content. Next, the endosomal membrane undergoes secondary invagination, inducing ESEs to shape intracellular multi-vesicles bodies (MVBs). When the MVBs merge with the cell membrane, exosomes finally take shape through exocytosis 38.

**Figure 1:**
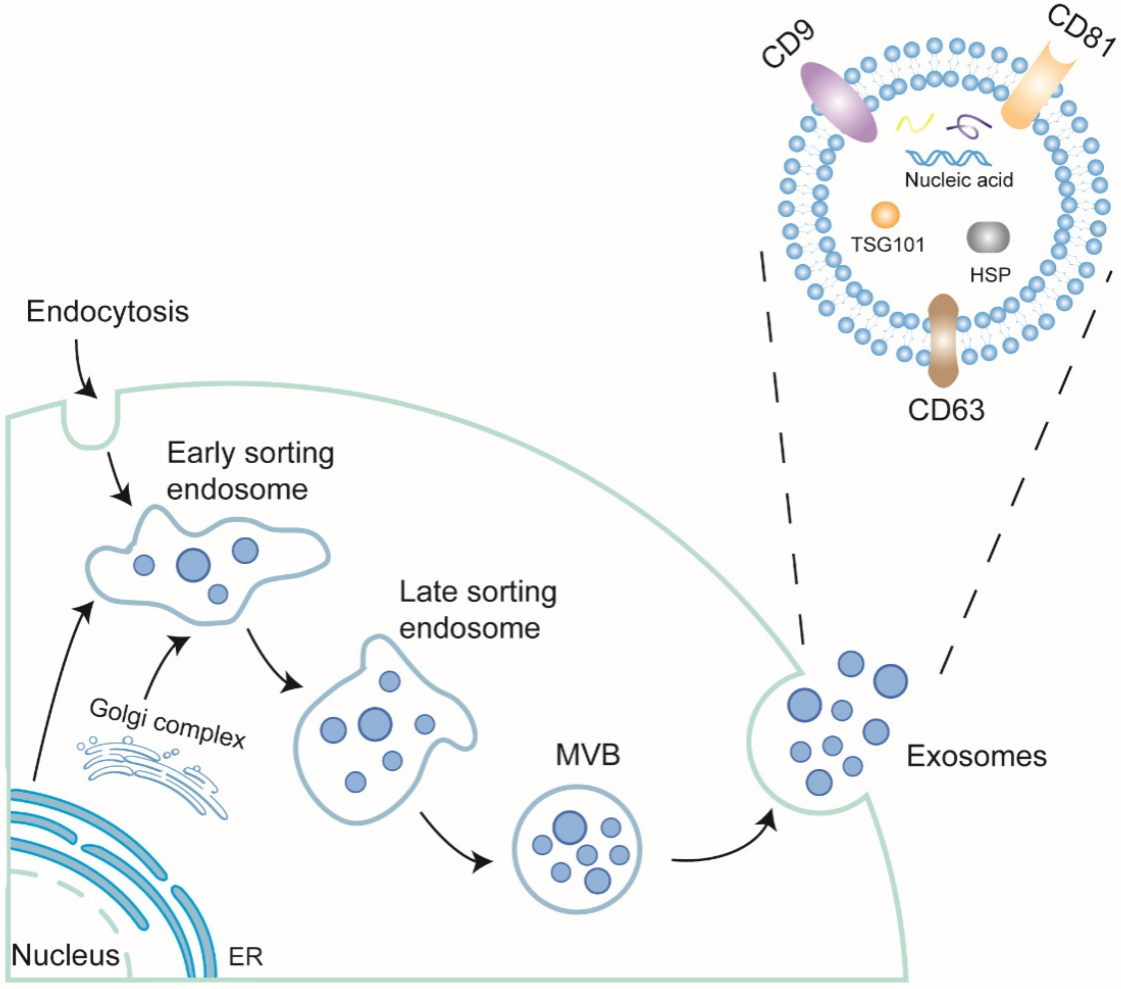
The biogenesis and structure of exosomes.The plasma membrane firstly invaginates into early sorting endosome (ESE) with the assistance of Golgi complex and endoplasmic reticulum. Then, ESE can generate late sorting endosome (LSE). The LSE undergoes second invagination and forms multivesicular body (MVB). MVB contains a structure called Intraluminal vesicles (ILV), which will be modified further. When MVB fuses with plasma membrane, ILVs will be released. At the time, ILV become exosomes. Exosomes are comprised of lipid bilayer and contents. The contents include nucleic acids (RNAs and DNAs), lipids and proteins. Some proteins are located on the membrane as receptor signals or receptors, some transverse the bilayer, like tetraspanin family, as hallmarks of exosomes, while some are in the exosomes, like TSG101, Alix, also function as hallmarks or play some certain roles.

The exosomes are formed by a phospholipid bilayer and bioactive cargos such as nucleic acids, including mRNAs, microRNAs (miRNAs) 39, circular RNAs (circRNAs) 40, long noncoding RNAs (lncRNAs) 41, Piwi-interacting RNAs (piRNAs) 42, double-stranded DNAs (dsDNA) 43, and tRNA-derived small RNAs (tsRNAs) 44, lipids, and proteins. Of these contained nucleic acids, piRNAs are novel compared with others and have been discussed its role in CVD 45. The lipids of exosomes, including cholesterols, ceramides, sphingomyelins, phosphatidylglycerols, and phosphatidylserine 46, 47, are largely derived from the parental cell and the Golgi complex. The proteins of exosomes include a series of tetraspanins, such as CD9, CD63, and CD81, some adhesion molecules and integrins, modulating signal transduction between cell-cell communication, of which the tetraspanins family, TSG101 and Alix has been regarded as common biomarkers for exosomes (Figure 1).

## Exosomes as diagnosis biomarkers for DIC

Due to the non-selective effects of DOX on cells, cardiotoxicity is unavoidable. Clinicians usually monitor cardiotoxicity by atypical symptoms (nausea, vomiting, and fever) and typical symptoms (hypotension, thrombosis, arrhythmia, myocarditis, and pericarditis). They also utilize techniques, such as echocardiography, multigated acquisition scanning (MUGA) 48, 49, and cardiac magnetic resonance (CMR) 50 to assess the heart injury induced by DOX. MUGA is the “gold standard” for supervising anthracycline-related heart damage 51, but its use is limited due to the risk associated with the radiation exposure. Advantageously, CMR identifies microstructure heart lesions, like diffuse fibrosis and anthracycline-related myocardial fibrosis 52-54, however, patients with pacemakers or other metals in body are excluded, limiting its use in clinic. In general, the evaluation of LVEF is not a sensitive tool because few changes occur in systolic function until significant myocardial damage has taken place, while MUGA is not safe and CMR is not universally applicable.

Cardiac troponins are routinely regarded as sensitive biomarkers for diagnosing acute myocardial infarction (AMI) 55. Also, elevated serum troponins are observed in a wide spectrum of cardiac injuries, such as cardiac trauma, CHF, pulmonary embolism, and chemotherapy-related cardiac toxicity 56. Some clinical studies have confirmed that troponins can be used to assess the cardiotoxicity induced by chemotherapy, specially trastuzumab and several tyrosine kinase inhibitors 57-59. Similarly, natriuretic peptide (NP) including brain natriuretic peptide (BNP), atrial natriuretic peptide (ANP), and their N-terminal counterparts (NT-pro BNP and NT-pro ANP), used to be considered as biomarkers for heart failure, have been explored as predictors of anticancer drug-induced cardiotoxicity 60-62. Both troponins and NP family can reveal heart damage regardless of various causes, but they not specific enough for DIC. Here, we introduce exosomes as promising, specific, and stable biomarkers for early cardiotoxicity induced by DOX.

### Exosomal miRNAs as biomarkers for DIC

Studies have confirmed that miRNAs in circulation could be markers for DIC 63. Here, we focus on exploring the potential of exosomal miRNAs for diagnosing DIC.

In a trial, EV-derived miRNAs in serum were found to express differently after single-DOX chemotherapy in dogs model of sarcoma compared with the initial. In detail, exosomal miR-107 and miR-146a were greatly downregulated, while exosomal miR-502 was upregulated. The changes in the pattern of exosomal miRNAs were more persistent compared to cTnI in serum 64. This results uncovered that exosomal-502 may be a damage marker for DIC, but it still needs to be validated following larger samples.

### Exosomal proteins as biomarkers for DIC

Studies have confirmed that exosomes contain many proteins related to cancers. And they could serve as biomarkers for pancreatic cancer 65, non-small cell lung cancer (NSCLC) 66, and gastric cancer (GC) 67. Besides, Chontida et al. reported that PYGB (brain/heart glycogen phosphorylase) in EVs, released from DOX-injured cardiomyocytes, could be a sensitive and early biomarker for DIC, for it started to elevate before troponin in the DOX-treated group 68. More proteins in exosomes or EVs await for further exploration as biomarkers for DIC (The part of exosomes functioning as biomarkers were shown in Table 1).

## Exosomes as therapeutic delivery vehicles

**Figure 2:**
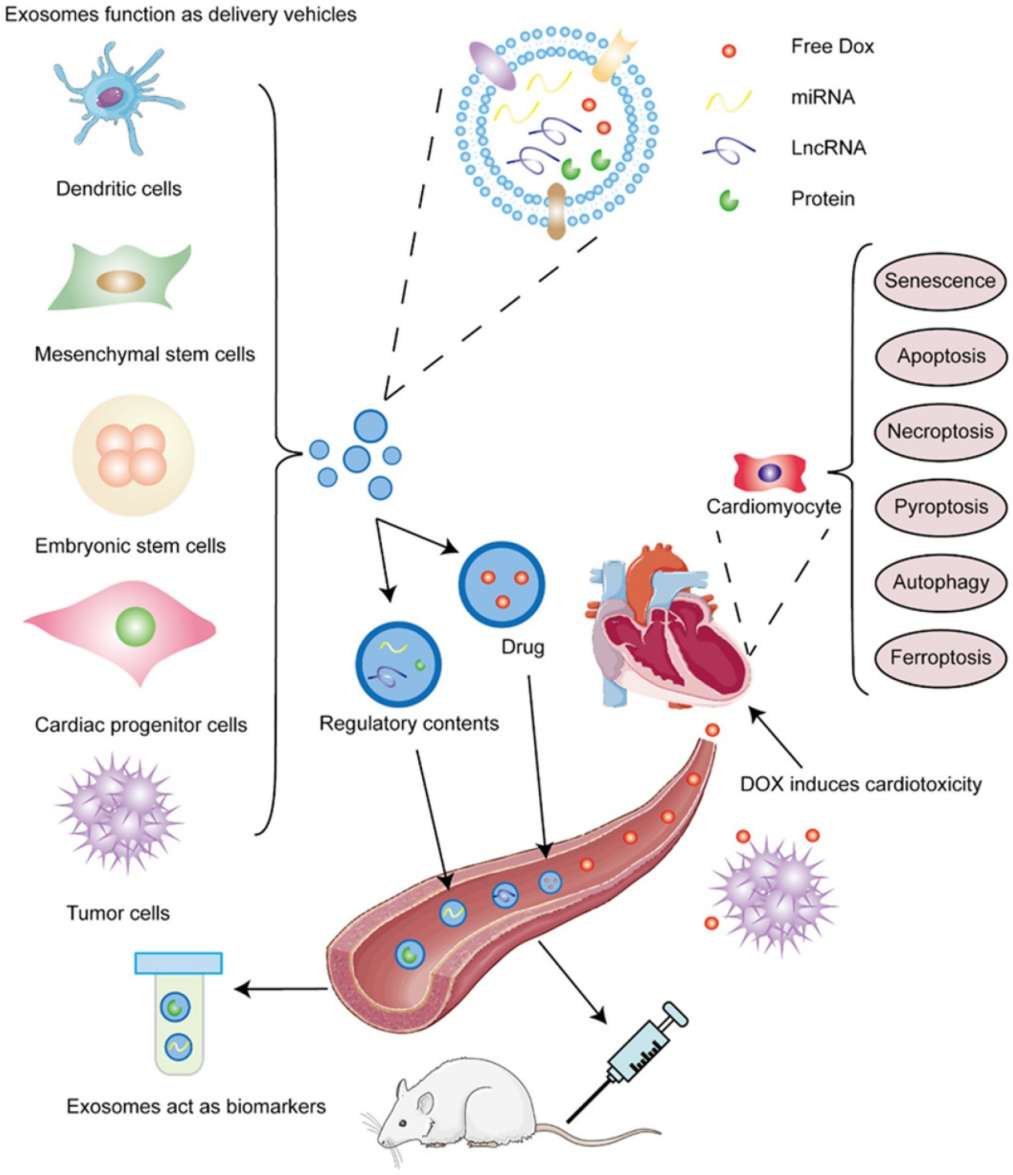
** Different sources of exosomes and contained cargos alleviate DIC. DOX induces cardiotoxicity.** DOX not only kill tumor cells through circulation, it but also damages cardiomyocytes at the same time and induces senescence and various death forms of cardiac cells, such as apoptosis, necroptosis, autophagy, pyroptosis, and ferroptosis. **Exosomes act as biomarkers for DIC.** Exosomes are comprised of phospholipid bilayer and contents, including proteins, which are also inserted into bilayer, and nucleic acids. MiRNAs and some proteins in exosomes can be used to forecast the emergence and prognosis of DOX-induced cardiotoxicity. **Exosomes function as delivery vehicles for DIC.** Varieties of cells, such as dendritic cells, mesenchymal stem cells, embryonic stem cells, cardiac progenitor cells, and tumor cells can secret exosomes or transfer contained miRNAs and lncRNAs to alleviate DOX-induced apoptosis and pyroptosis, decreasing DIC.

The superior characteristics of non-immunogenicity and high-capacity make exosomes ideal vehicles for chemotherapeutic drugs and genetic regulators. In this section, we introduce cells that produce exosomes and discuss the bioactive contents delivered by them to alleviate cardiotoxicity. Summaries are shown in Table 2 and Table 3. Schematic presentation is shown in Figure 2.

### Exosomes derived from different cells to alleviate DIC

Cell transplantation has been explored to exert repair functions through injection, but its application is limited for low survival rate and immunogenicity *in vivo*. Increasing evidence has shown that exogenous cell transplantation play therapeutic function through paracrine of EVs, since it is reversed by exosome inhibitor, GW4869 76, 77. Thus, experts have pointed out that EV/exosomes-based injection could replace cell transplantation, which retain the therapeutic effects and are more safe and easy to store compared with cell transplantation.

#### Embryonic stem cell-derived exosomes

As is mentioned before, pyroptosis is a kind of cardiomyocyte death induced by DOX. This death is characterized by the release of vast pro-inflammatory factors and participates in the development of infectious diseases, neurological disorders and cardiovascular diseases (CVD). NLRP3, which has been validated its function in endothelial dysfunction 78, plays a key role in pyroptosis and is considered as a marker for it. Exosomes derived from embryonic stem cells were demonstrated to inhibit DOX-induced pyroptosis with the decrease of NLRP3 and other pyroptosis-related indexes 69. Similarly, Dinender and his colleagues also found that this kind of exosomes could repress the expression of some pyroptosis markers and reduce cardiac hypertrophy, improving heart functions 70. These studies verified the potential of embryonic stem cells as reliable sources of exosomes for attenuating DOX-induced pyroptosis.

#### Mesenchymal stem cell (MSC)-derived exosomes

Mesenchymal stem cells (MSCs) mainly derive from bone marrow and adipose tissues and have the potential of proliferating and differentiating into multiple cell types. Researchers have used mesenchymal stem cells (MSCs) as common, efficient, and productive sources of exosomes, for they possess the capacity to promote osteogenesis 79, alleviate inflammation 80, treat cerebrovascular diseases 81, 82, and heart diseases 83, 84. There was a comparison between the therapeutic effects of human adipose-derived mesenchymal stem cells (AT-MSCs) and bone marrow mesenchymal stem cells (BM-MSCs) for DIC on rats, the results displayed that they two were equal in alleviating cytotoxicity through decreasing inflammation responses and fibrosis 85. Moreover, hypoxia, used to be considered as a detrimental factor for it can induce ischemia-reperfusion (I/R) injury, has also intriguingly improved biological activity and showed better therapeutic functions. For instance, human adipose-derived mesenchymal stem cells (AT-MSCs) were treated with hypoxia and stimulated the secretion of exosome^Hypoxia^, exosome^Hypoxia^ showed superiority in protecting cardiomyocytes from senescence induced by DOX 71. The indistinguishably therapeutic effects of AT-MSC and BM-MSC implied that the secretion of exosomes was the common characteristic of MSCs, and the pretreatment of hypoxia could achieve better effects.

#### Cardiac progenitor cell (CPC)-derived exosomes

Exosomes derived from CPCs have been proven to be cardioprotective for myocardial infarction (MI) and ischemia/reperfusion (I/R) *in vitro* and *in vivo* 76, 77. In another study, exosomes derived from CPCs were isolated from conditioned medium and pretreated neonatal rat ventricular myocytes, then they were exposed to Doxorubicin and trastuzumab (DOX/Trz) in succession. The results displayed that the exosomes ameliorated DOX-/Trz-induced cardiotoxicity through repressing inflammatory responses and decreasing myocardial fibrosis. Notably, exosomal miR-146-5p partly mediated the cardioprotection 72. This result was similar to a previous study, which found that miR-146a in exosomes inhibited oxidative stress-induced apoptosis of cardiomyocytes and decreased the size of in farction 77. MiR-146 present in exosomes have exhibited preeminent regeneration ability.

#### Tumor cell-derived exosomes

Usually, experts explore the possibility of making exosomes target tumors more selectively, actually, tumor itself is a source of exosomes. Previous studies have confirmed the “homing behavior” of stem cells. This behavior could guide stem cells to reach the impaired cells or organs and repair them. If tumor cells have similar behavior, exosomes derived from tumor cells may kill themselves locally without distant movement. Recently, Li and co-workers discovered that tumor-derived exosomes preferentially fuse with parent cancer cells *in vitro*, this cancer-homing behavior similar to the Trojan horse enables such exosomes to be better therapeutic agent to treat cancer and enhance the therapeutic drug retention 73. However, it remains an obstacle that exosomes can be rapidly swallowed up by the mononuclear phagocyte system, this characteristics hindered them to be delivered into tumor. Thus, Wan et al. designed a two-step exosome delivery strategy, in short, they knock down endocytosis-related gene, *Cltc*, in exosomes and successfully blocked the uptake of exosomes from MPS, facilitating exosomes to distribute more efficiently 86. However, tumor-derived exosomes also promote the metastasis of tumors. Exosomes play the role of immune activation and suppression at the same time. The activation effect mainly depend on its role of antigen presentation, while the suppression effect mainly depend on the ligand they carry 87. So the effects of various ligands in exosomes should be identified so as to achieve therapeutic goal and avoid tumor progression.

#### Dendritic cell (DC)-derived exosomes

Dendritic cells (DCs) are the professional antigen presenting cells. They secrete EVs to play a significant role in CVDs 88-90, tumors 91-93, myasthenia gravis 94, and transplant organ research 95.

Natural exosomes are regarded as less immunogenic and toxic, they are not always therapeutic. For example, immature dendritic cells (imDCs) can produce exosomes, but they could not target tumor selectively, when they were engineered to express a special exosomal membrane protein called *Lamp2b*, they could deliver cargo of DOX to tumor selectively and inhibit their growth without obvious cardiotoxicity, which is of great potential for clinical application 74. Recently, Phung et al. developed an anti-cancer strategy based on DC-derived exosomes: anti-CTLA-4 antibody was anchored in lipids of exosomes of DCs, which guide the exosomes towards T cells, in addition, they endowed the exosomes with ability to block checkpoint, both enhancing the T cell response to cancer cells synergistically 75. Stimulating cancer-specific T cells to response is expected to become a replacement strategy for cancer vaccine and can also decrease the usage of DOX, thus ameliorating cardiotoxicity.

### Delivery vehicles of different cargos

#### Delivery of DOX

As is mentioned above, the non-targeted damage has limited the use of DOX in cancer patients. Hence, it is necessary to find a strategy to make the drug more tumor-specific. Increasing studies have proven that exosomes can be constructed to deliver DOX to treat cancers and they showed better loading capability and splendid efficacy compared to the traditional form of DOX 96, 97. In order to compare the cardiotoxicity between DOX encapsulated in exosomes (exoDOX) and free form, tumor volume was measured after treatment of them to assess the killing effect, the results showed that exoDOX was potent as free DOX and nontoxic meanwhile 98. Similarly, exosomes isolated from breast cancer cells and ovarian cancer cell lines in the mouse models, respectively, were loaded with DOX, they also maintained the efficacy of DOX without enhancing the cardiotoxicity of DOX 99. Exosomes have become common delivery vehicles of DOX to target various cancers and receive better therapeutic effects than free DOX.

#### Delivery of regulatory genes

##### Delivery of miRNAs

MicroRNAs, 19~22 nucleotides, can inhibit mRNA translation or promote degradation, thus negatively regulating expression of genes 100. They participate in the occurrence and development of heart diseases and regulate cardiac function, such as heart morphogenesis and growth, myocardium contraction, and conductance of electrical signals, thus imparting therapeutic effects 101-103.

Since DOX induces cardiomyocytes death and cardiac atrophy, promoting the proliferation of cardiomyocyte is an approach to reversing damage 104. Researchers demonstrated that cardiac fibroblast-derived exosomes transported miR-21 to mediate the hypertrophy of cardiomyocyte and they were potential therapeutic candidates for DOX-induced atrophy 105. Chemoresistance not only increase the cardiotoxicity as the dosage accumulates, but also cause chronic inflammation. Relieving the inflammation may be conducive to solving this problem. Specially, chemoresistance is more common to occur during or after breast cancer. So, addressing breast-related cardiotoxicity is of great significance. Deng and colleagues identified that DOX treatment in mouse model of breast cancer resulted in the production of myeloid-derived suppressor cells (MDSCs), which secreted exosomal miR-126 to promote lung metastasis and reverse DOX-induced death of MDSC, developing inhibitors for exosomal miR-126 secreted by MDSCs may be a target for solving DOX-resistance in breast cancer 106.

Triple-negative breast cancer (TNBC) refers the Results of immunohistochemical examination of estrogen receptor, progesterone receptor and proto-oncogene (*Her 2*) are all negative. Its prognosis is poor in breast cancer and deserves attention. Gong and coworkers identified that a kind of exosomes could express disintegrin and metalloproteinase 15 on the membrane (A15-Exos), which can deliver DOX and miR-159 modified by cholesterol to TNBC cells at the time, exerting synergetic therapy of anti-cancer through silencing *TCF-7* without adverse effects 107. For addressing chemo-resistance in hepatocellular carcinoma, Lou et al. isolated exosomes from adipose tissues-derived mesenchymal stem cells (AMSCs) and constructed miR-199a-modified AMSCs (AMSC-199a) through lentivirus injection of miR-199a. They found that AMSC-Exo-199a administration effectively sensitized HCC to DOX by targeting *mTOR* pathway 108. Treatment failure of TNBC in clinical practice is mostly due to chemo-resistance and metastasis. Researchers found that miR-770 in exosomes derived from THP-1 cells can decrease chemo-resistance of DOX via promoting apoptosis of tumor cells in TNBC, and miR-770 was elevated in tissues-sensitive to chemotherapy evidenced this result clinically, displaying that exosomal miR-770 may be a therapeutic target or prognostic indicator for TNBC 109.

For other chemo-resistant cancers, researchers found that miR-501 were enriched in exosomes derived from DOX-resistant cell line of gastric cancer, it plays a role in inhibiting the apoptosis of gastric cancer cell and promoting it to proliferate and migrate through *Akt* pathway 110.

The role of miRNAs are more studied in exosomes for its abundance in exosomes compared with other nucleic acids. But other nucleic acids should not be ignored, they may be more crucial in modulation than miRNAs though less.

##### Delivery of lncRNAs

Long-noncoding RNAs (lncRNAs), length exceeding 200 nt, are a subset of non-coding RNAs and modulate transcription and expression of genes from short- and distant range. Increasing evidence points out that lncRNAs are involved in cardiomyocyte proliferation, cardiac repairing and senescence 111, 112.

Recently, lncRNA H19 contained in exosomes were found to regulate DOX resistance of breast cancer. The treatment of exosomal H19 from DOX-resistant breast cells sensitive cells increased chemoresistance, while this phenomenon could be reversed by downregulating H19. This implied that exosomal lncRNAs could affect DOX resistance and act as a therapeutic target 113. As is mentioned above, exosomes generated from MSCs could inhibit DOX-induced cardiac senescence, this cardioprotective effect was mediated by overexpressing lncRNA *MALAT1* in exosome^Hypoxia^. In detail, *MALAT1* sponged microRNA-92a-3p, and the binding of them activated *ATG4a*, improving mitochondrial metabolism and thus partially playing a cardioprotective role in DOX-induced cardiac lesions 71.

##### Exosomal proteins modulating DIC

Proteins are the inherent structure of exosomes. And certain proteins of exosomes can mediate communication between exosome-secreting and recipient cells. They may have an effect on the development and treatment of DIC.

Recently, Tania et al. attested that EVs membranes contain a special protein called gap junction protein Connexin 43 (CX43), a variety of channel of exosome. It could facilitate the contents of EVs to enter into recipient cells 114. These traits may enable this protein to aid EV to aim at the target more selectively. Recognition of checkpoints for killing cancer cells by immunotherapy has attracted much attention. CD47, a cell surface glycoprotein, is a novel checkpoint commonly express on the surface of cancer cells. Tumor cells secrete exosomes highly enriched CD47, which promoted metastasis and facilitated a microenvironment for tumor progression. Studies have demonstrated that anti-CD47 can achieve better therapeutic effects 115. It has been reported that there was mutual effect between CD47 and SIRPα, a signal protein on phagocytic cells. The binding of them prevented phagocytic cells swallowing tumor cells. However, exosomes harbored with SIRPα variants (SIRPα-exosomes) could break the association between CD47 and SIRPα and relieve the resistance of phagocytic cell, which provides a strategy for enhancing immune response to cancers based on exosomal proteins 116.

## Conclusions and Future Perspectives

**Figure 3:**
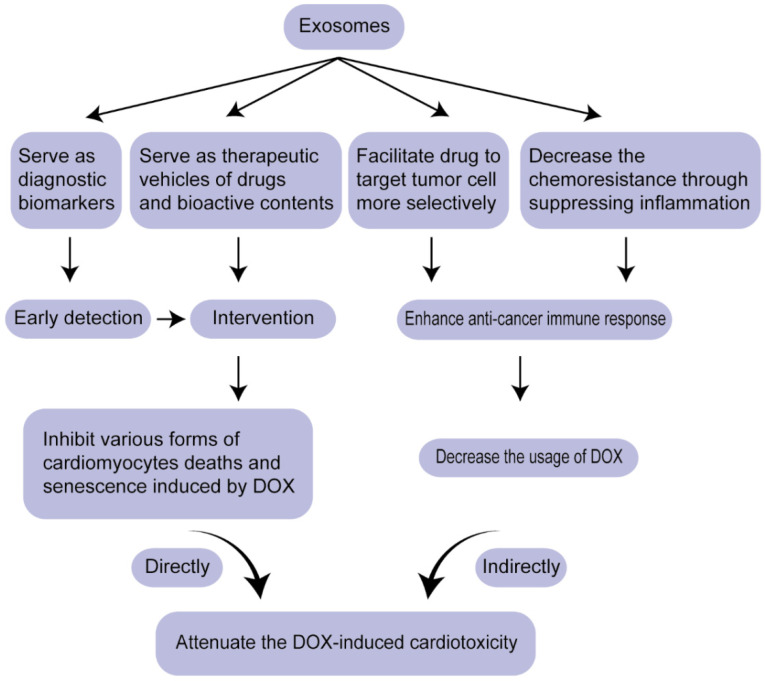
Overview of the strategy for attenuating DIC based on exosomes.

CVDs and cancers are the top two fatal diseases worldwide. Traditionally, they are viewed as independent diseases. With the wide application of chemotherapy and the emergence of heart symptoms, accumulating attention was attracted on how to mitigate heart reverse effects, cardiotoxicity, without weakening the potency of drugs, contributing to the birth of a novel discipline-CardiOncology. Exosome, a kind of liquid biopsy, mitigate DIC via two pathways: serving as diagnostic biomarkers for early detection and therapeutic vehicles for drugs or regulatory molecules (nucleic acids and proteins), which not only regulate various forms of cardiomyocyte deaths induced by DOX and alleviate cardiac injuries directly, but also modulate chemo-resistance of cancer cells, thus decreasing the cardiotoxicity indirectly (Figure 3). miRNAs and proteins in exosomes as biomarkers for DIC have been discussed, the potential of lncRNAs, circRNAs and other nucleic acids contained in exosomes needs to be explored further as reliable biomarkers for cardiac toxicity. In the aspect of therapeutic vehicles, exosomes as anti-tumor vaccination have come into phase I trial. Exosomes can be expected as a replacement of anti-tumor vaccination to enhance the identification and killing response mediated by immune cells, thus decreasing the dosage of DOX and attenuating cardiotoxicity.

There are still some problems. First, means of enriching exosomes require further improvements. Second, more tools need to be developed to facilitate exosomes to target tumor more selectively besides CRISPR/Cas9 system 117. Third, how to make exosomes stably exist in circulation is also a problem. Recently, researchers have developed a method detecting breast cancer through amplifying mRNAs in urine 118, which evoke researchers to find more available biomarkers for DIC in other body fluids.

Collectively, due to their advantageous features discussed in this review, exosomes will be preeminent in the aspects of diagnosis and therapy for DIC in the near future with the development of proteomics, genomics, and high-throughput sequencing technology. Combining the superior characteristics of exosomes and nanoparticles designed for capturing biomarkers or delivering mRNA and siRNA 119, will bring favorable diagnostic and therapeutic effects.

## Figures and Tables

**Table 1:** Exosomes/EVs as biomarkers for diagnosing DIC

Parts of exosomes/EVs	Components	Model	Distribution	Significance	Ref.
miRNAs	miR-502	dog	serum	Uncovered the potential of miRNAs as biomarkers	64
	miR-107/146a				
protein	PYGB	mouse	serum	Detected heart injury early and sensitively	68

**Table 2:** Exosomes released by different cells alleviate DIC

Parent cell	Recipient cell	Effects	Ref.
Embryonic stem cell (ESC)	H9C2 cells	Inhibit pyroptosis	69
		Inhibit pyroptosis	70
Adipose-mesenchymal stem cell (MSC)	Cardiomyocyte	Protect cardiomyocyte from senescence	71
Cardiac progenitor cell (CPC)	Cardiomyocyte	Repress oxidative stress and decrease myocardial fibrosis	72
Tumor cell	Tumor cell	Target tumor selectively	73
Dendritic cell (DC)	Tumor cell	Inhibit tumor growth	74
	T cell	Facilitate T cell response to cancer	75

**Table 3:** Different cargos released by exosomes regulate DIC

Cargos	Classfications	Secreted cell and detailed cargo	Recipient cell	Effects	Ref.
DOX	NA	NA	Breast cancer cell	Increase DOX efficacy of killing tumors	99
Non-coding RNAs	miRNAs	Myeloid-derived suppressor cells (MDSC)-derived miR-126	Tumor cell	Inhibition of exosomal miR-126 induce the death of MDSC	106
		Monocytes-derived miR-159	Breast cancer cell	Lead to cancer cell death	107
		Adipose-MSC-derived miR-199a	Hepatocellular carcinoma (HCC)	Promote the death of HCC	108
		Breast cancer tissue-derived miR-770	Triple negative breast cancer (TNBC)	Induce the apoptosis of tumor cells	109
		miR-501	Gastric cancer(GC)	Inhibit the apoptosis of GC	110
	lncRNAs	Breast cancer-derived lncRNA H19	Breast cancer cell	Resist the apoptosis of cancer cells	113
		Mesenchymal stem cell (MSC)-derived lncRNA MALAT1	Cardiomyocyte	Protect cardiomyocyte from senescence	71
Protein	CD47	Tumor cell	Tumor cell	Anti-CD47enhances DOX-induced tumor death synergistically	115
